# Improved interprofessional communication, handover and ward rounds in critical care (ICARUS)

**DOI:** 10.1186/cc14604

**Published:** 2015-03-16

**Authors:** P Hopkins, A Chan, S Rasoli, C Bell, K Peters, A Feehan, J Dawson

**Affiliations:** 1 King's Health Partners AHSC, London, UK

## Introduction

There is growing evidence that optimising communication and patient assessment practices including ward rounds and handoffs can improve clinical, safety and operational outcomes, particularly in the critical care setting [1,2]. Here we describe the design and baseline phases of a 5-year project utilising improvement sciences to optimise the quality of interprofessional communication, handoffs and rounding in one of the largest critical care units in the UK.

## Methods

We obtained institutional ethical and research approvals. We used mixed methods including interviews of opinion leaders and a representative cross-section of staff, roundtables, a survey targeting the whole critical care team (*n *= 546) and a Delphi exercise to generate a baseline consensus for the need to improve and a set of novel quality improvement interventions and tools. We tested two of these in a pilot plan-do-study-act (PDSA) cycle.

## Results

Baseline consensus for the need and potential to improve was obtained (97.4% and 94.5%). Despite a large degree of heterogeneity of perceptions around current communication and rounding practices, it was possible to develop a set of interventions based on consensus that could be applied in this complex setting. These included a handoff bundle, an operational touch-base, a unit-level safety briefing, a unit-level safety check, a lean rounding bundle and board rounds. These core interventions were supported by several more detailed resources describing the evidence base around best handoff and rounding practices; and a feedback document that described all outputs and recommendations from the ICARUS project. A pilot PDSA cycle demonstrated a 55.3% and 76.3% improvement in key information transfer using a safety briefing and board round summary.

## Conclusion

Despite wide heterogeneity in baseline beliefs, opinions and perceptions around inter-professional communication, rounding and handoffs, we were able to develop a novel set of feasible quality improvement interventions, targeting these areas, that can be applied in a large complex critical care setting. Furthermore, they can be driven by improvement science methodology and tested for effectiveness using qualitative and quantitative measures. We now plan to use these interventions to deliver quality improvements in communication practices in parallel with the planning and implementation phases of a new critical care facility and electronic clinical information system.
